# Covalent Modulation of Protein Misfolding and Aggregation Processes in the Context of Neurodegenerative Diseases

**DOI:** 10.1002/ardp.70285

**Published:** 2026-07-03

**Authors:** Filippo Basagni, Elena Roggiolani, Anna Minarini, Michela Rosini

**Affiliations:** ^1^ Department of Pharmacy and Biotechnology Alma Mater Studiorum—University of Bologna Bologna Italy

**Keywords:** antiaggregating compounds, covalent inhibitor, intrinsically disordered proteins, neurodegenerative diseases, proteinopathies

## Abstract

Misfolded protein aggregates represent major histopathological hallmarks of neurodegenerative diseases, differing in the structural components and brain regions affected. Furthermore, the formed assemblies act as key players in developing and fostering neurotoxic processes, with distinct mechanisms depending on the stage of the amyloid cascade. Particularly, the oligomer intermediates are now considered as the main drivers of neurotoxicity, thus requiring an early antiaggregant therapeutic intervention to achieve a significant neuroprotective efficacy. Among different strategies, direct interaction at early stages preventing aggregation is quite intricate due to the considered undruggability of misfolded monomers. In this context, a covalent approach targeting specific functional nucleophilic residues within disordered proteins can offer an intriguing opportunity to overcome these weaknesses. Therefore, in this review, we outline covalent modulators of misfolding and aggregation processes reported to date, referring to the major misfolded proteins in the neurodegenerative context (*i.e*., β‐amyloid, tau, α‐synuclein, and superoxide dismutase 1) to highlight their potential both as valuable pharmacological tools or therapeutic perspectives.

## Graphical abstract

This review highlights strengths and pitfalls regarding the covalent inhibitors of misfolded protein aggregation processes in the context of neurodegenerative diseases, with the aim to enhance their potential role as valuable pharmacological tools and prospective therapeutics.

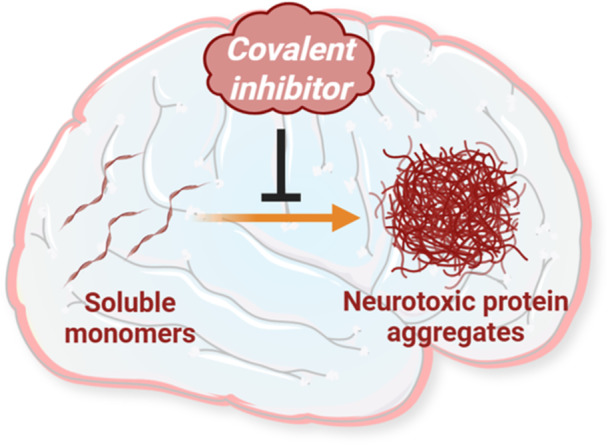

**Abbreviations**
α‐Syn: α‐synuclein
Aβ: β‐amyloid peptide
AD: Alzheimer's disease
ALS: amyotrophic lateral sclerosis
APP: amyloid precursor protein
BBB: blood–brain barrier
BQ: benzoquinone
CA: cinnamaldehyde
CCS: copper chaperone for SOD1
CNS: central nervous system
DLB: dementia with Lewy bodies
EC: epicatechin
EGCG: (‐)‐epigallocatechin‐3‐gallate
MBD: microtubule‐binding domain
MS: mass spectrometry
MSA: multiple system atrophy
NFT: neurofibrillary tangles
NMR: nuclear magnetic resonance
PD: Parkinson's disease
PHF: paired helical filament
POM: polyoxometalate
PrP: prion protein
SOD1: superoxide dismutase 1

## 1 Introduction

In the physiopathology and etiopathology of the majority of neurodegenerative diseases, formation and deposition of neurotoxic aggregates of misfolded proteins play a pivotal role [1]. However, the mechanism by which this happens is still unclear. Each neurodegenerative disease is characterized by a different distribution and composition of protein aggregates, although they share similar morphological characteristics and triggering events [2]. Since Alzheimer's findings on extracellular amyloid plaques and intracellular neurofibrillary tangles (NFTs) in post‐mortem brain of Alzheimer's disease (AD) patients, aggregates of misfolded proteins have represented the typical pathological hallmarks of almost all neurodegenerative disorders: from Lewy bodies in Parkinson's disease (PD), to huntingtin deposits in Huntington's disease (HD), aggregates of superoxide dismutase 1 (SOD1) in amyotrophic lateral sclerosis (ALS) or prion proteins (PrP) in some forms of encephalopathies [3]. Genetic factors, such as mutations in protein primary structure, can favor destabilization of physiological conformation and induce misfolding. Parallelly, environmental factors including pH, oxidative stress, dyshomeostasis of metal ions or pathological chaperones proved to be able to catalyze aggregate formation [4].

**Figure 1:**
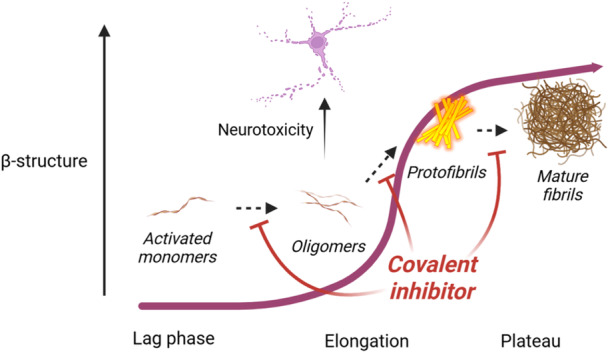
Step‐wise process of misfolded protein aggregation and putative intervention points of covalent inhibitors.

Although the pathophysiology and symptoms for this type of proteinopathies vary with the nature of misaggregated proteins and affected brain region or cells' population, the primary cascade leading to the formation and deposition of misaggregated proteins is highly similar [5]. In most cases, native monomeric protein is predominantly composed of α‐helical and unordered structures, whereas misfolded polymers are rich in β‐sheet conformation. Different starting pathological triggers (*i.e*., altered post‐translational modification, proteolytic cleavage, oxidative stress) promote the formation of aggregating‐prone monomers, which are commonly refolded into β‐sheet structures. These misfolded units self‐associated, building up the primary nucleation seeds that, by means of an iterative cycle, lead to oligomers, protofibrils, and then fibrils (Figure 1) [6]. Kinetic studies suggested that the critical event is the formation of protein oligomers, which consequently act as seeds to propagate nucleation‐dependent polymerization in a similar manner to crystallization. It has been found that the probability of aggregation is directly related to increasing protein concentration, as it happens in genetic mutations that lead to local altered protein dosages [7].

Nowadays, it is affirmed that intermediates of misfolded proteins at different stages exert diverse toxic effects. Particularly, misfolded oligomers could activate signaling pathways leading to apoptosis, while bulky aggregates break down neuronal connections as well as participate in recruiting immune cells and activate inflammasomes [8]. Most importantly, mid‐term soluble oligomers are now considered the main drivers of neurotoxicity [9, 10, 11]. Based on these premises, it seems much more appropriate to have an ideal early intervention preventing the misfolding process or monomer aggregation rather than targeting already formed aggregates. Unfortunately, this clashes with the lack of early reliable biomarkers that could allow a prompt therapeutic intervention [12].

Although the complexity of the multi‐layered neurotoxic cascades, several small molecules were developed to reduce cytotoxic aggregates levels through modulation of oligomers production, inhibition of their aggregation and enhancement of the clearance. Through this review we would highlight the upsides and pitfalls of aggregation inhibitors acting with a covalent mechanism of action to elucidate their potential role both as therapeutic agents or pharmacological investigation tools. Particularly, given the electrophile‐sensitive behavior of “activated” monomers, they bear several functional nucleophilic residues (*i.e*., mainly cysteines or lysines) that can be covalently trapped with potential modulatory effects at different aggregating stages (Figure 1) [13]. Therefore, after properly introducing and briefly outlining the aggregation processes and the pathological context of each targeted protein (*i.e*., β‐amyloid, tau, α‐synuclein and SOD1), notable examples of the associated covalent modulators will be reported to deliver a glimpse on the realm of different covalent approaches exploited toward the main disordered proteins involved in neurodegenerative diseases. At this stage, we focused our attention on covalent approaches developed toward the aforementioned amyloidogenic proteins implicated in neurodegenerative diseases acting in the central nervous system because they have been widely dissected to these purposes and perfectly highlight the potential of trapping functional nucleophilic residues in the context of misfolding‐related pathologies. Notably, the same approach can be shifted, or has already been applied, to other pathogenic proteins that characterize diseases either inside or outside the central nervous system [14, 15].

## 2 β‐Amyloid Peptide

Aβ peptides are the main component of neuritic plaques, one of the major hallmarks in AD histopathology, and are always considered as the main driver in AD pathogenesis and development. The so‐called “amyloid hypothesis” dominated the gold rush toward new effective therapeutics for AD treatment, leading to plenty of failures and some recent successes (*e.g*., monoclonal antibodies lecanemab and donanemab), which forced a reflection about the actual role of Aβ in AD etiopathology and its suitability as therapeutic target [16, 17]. For example, significant gaps were not reported regarding the number of senile plaques in healthy and aged/dementia subjects, also lacking correlation between plaques burden and disease severity, while considering NFT loading or Aβ/tau synergy seems more reliable [18, 19].

Aβ is derived from the proteolytic cleavage of amyloid precursor protein (APP), an integral membrane protein mainly expressed at synapses in neurons and exerting physiological trophic functions in the CNS [20]. The sequential cleavage by β‐ and γ‐secretases is required to produce Aβ, consisting of a 37–49‐residue peptide, among which the Aβ40 isoform is the most common, while Aβ42 represents the most aggregating‐prone one [21, 22, 23]. Normally, the non‐amyloidogenic cleavage of APP is predominant, whereas pathological triggers boost the concentration of Aβ leading to neurotoxic fibrillization and aggregation [22]. To note, Aβ at physiological concentrations exerts neuroprotection as well as synaptic plasticity, memory, and learning enhancement [24]. Conversely, impairment of Aβ clearance machinery paired with its pathological overproduction makes the Aβ monomer outbreak [25]. Besides lower solubility of Aβ42, there are several aspects of Aβ monomer homeostasis that can affect its aggregation: for example, glycation and *N*‐terminal truncation promote aggregation while oxidation at Met35 residue mitigates this tendency [21]. From monomer misfolding and stepwise aggregation originate oligomers characterized by β‐structure‐rich aggregation cores, protofibrils, and mature fibrils, which end up in building the neuritic plaques [26].

Within the realm of different Aβ‐therapeutic strategies, aggregation inhibitors represent only a small niche that turned out to be worthy to fully depict Aβ aggregating processes at the molecular level [25, 27]. Below, examples of Aβ‐directed antiaggregant inhibitors exerting a covalent mechanism are reported and categorized according to the respective chemical structure.

### 2.1 Polyphenols

Several natural and nature‐derived polyphenols proved to inhibit protein aggregation [28]. Their structure is generally associated with a wealth of biological properties, ranging from antioxidant, antinflammatory, and anticancer activities. Some of these are in clinical trials for neurodegenerative disease treatment (*e.g*., curcumin, resveratrol, and epigallocatechin‐3‐gallate) [27]. Regarding inhibition of Aβ aggregation, polyphenols have been demonstrated to prevent oligomer cytotoxicity and aggregation with different mechanisms of action based on their electrophilic characteristics.

**Figure 2:**
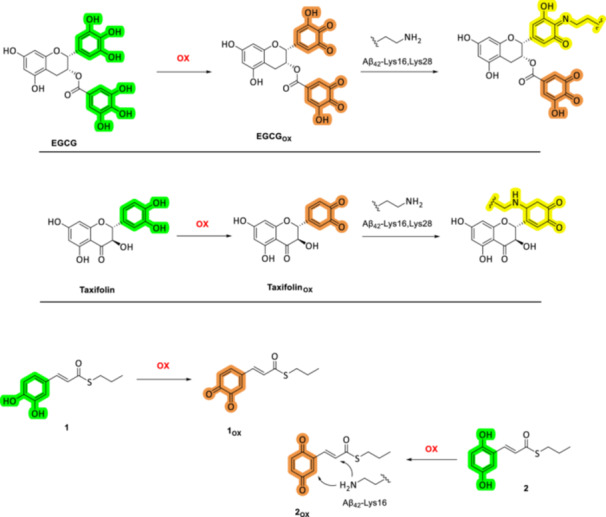
Polyphenols exerting Aβ antiaggregating activities based on a covalent mechanism.

Particularly, (‐)‐epigallocatechin‐3‐gallate (EGCG, Figure 2) has previously been demonstrated to covalently interact with the cysteine group of target proteins through autoxidation [29], while Aβ lacks this residue. Regarding Aβ_40_, it has been suggested that EGCG forms a putative Schiff base formation with Lys16 and/or Lys28, upon previous autoxidation into quinone form, albeit this interaction was confirmed to be not essential for the anti‐amyloidogenic activity [30]. Actually, in this case, the hydrophobic binding site engagement was suggested as the major driving force in mature amyloid fibrils remodeling with the resultant prevention of Aβ aggregation. As a consequence of this mechanism, EGCG demonstrated the ability to tackle toxic oligomers formation and turn mature fibrils into non‐toxic oligomers [31]. The same covalent‐targeting approach involving lysine residues was further confirmed with EGCG and other polyphenols for different aggregating‐prone proteins [32].

(+ )‐Taxifolin represents another catechol‐bearing flavonoid that inhibits Aβ_42_ aggregation [33]. Compared with other non‐catechol polyphenols, the anti‐amyloidogenic activity was mainly driven by hydrophobic interactions, but the pro‐oxidant properties of the catechol fragment with putative covalent adduct formation gave a boost in this respect. Upon autoxidation to *o*‐quinone, oxidized taxifolin covalently binds amyloid Lys16 and Lys28 through Michael addition (Figure 2) and, because these residues are situated in the intermolecular β‐sheet region, the resulting Aβ_42_ aggregates would be destabilized by the adduct formation, thus limiting plaque deposition. To note, taxifolin could suppress the elongation phase in the aggregation of wild‐type Aβ_42_, rather than the nucleation phase. In‐depth computational investigations further confirmed the perfect location of the oxidized flavonoid within the hydrophobic groove between Lys16 and Glu22 as an essential step preliminary to the nucleophilic attack of lysine [34].

In this context, in 2016, we reported a series of thioester polyphenols endowed with neuroprotective properties such as Nrf2 activation and Aβ aggregation inhibition at low micromolar levels. Notably, for the antiaggregating efficacy, the presence of a catechol moiety was essential, and the thioester group was preferred with respect to the ester or amide, confirming the importance of initial hydrophobic interactions [35, 36]. Therefore, we further investigated the electronic and redox properties of this scaffold for the purpose of this activity by simply shifting one hydroxy function in the aromatic core [37]. Only catechol and hydroquinone derivatives (**1** and **2** in Figure 2) resulted in efficient antiaggregants, and by means of electrochemical analyses, a correlation was found between the antiaggregating potency and the oxidation potential, thus suggesting the proelectrophilic character of this class of compounds as essential for the activity. Further mass spectrometry (MS) investigation revealed a different mechanism of action: reversible interactions for the catechol one and formation of a covalent adduct with Lys16 of Aβ_42_ for the *p*‐hydroquinone analog. Particularly, as emerged from the molecular weight of the formed adduct, compound **2**, upon oxidation, should undergo a double attack from nucleophilic Lys16, and quantum mechanical calculations proposed a starting anchorage at *p*‐quinone ring, which may subsequently evolve through an intramolecular cyclization at the vinyl position. Moreover, an oxidation‐based mechanism was also accounted for underlying the antiaggregating activity of compound **2**, albeit a detected cytotoxic profile required future chemical optimization.

In this context, Lim and colleagues attempted to rationalize the antiaggregating properties of redox‐active small molecules. Particularly, among a class of phenyls carrying Lewis basic functional group (*i.e*., amine, hydroxyl, carboxylate), only the redox‐active *para*‐substituted analogs were able to alter Aβ40 aggregation process by means of oxidation‐based mechanism or covalent adduct formation through the *in situ* formed benzoquinone (BQ) derivative. To note, benzene‐1,4‐diamine revealed superior *in vivo* efficacy in the AD mouse model as well as lower toxicity in respect to BQ, the product of its oxidative transformation [38]. A deeper study among positional isomers confirmed the *para*‐substituted compounds as more effective in vitro and in vivo modulators of amyloid plaque burden, and only 4‐aminophenol proved to engage Aβ40 with a covalent interaction under the BQ form, thanks to its peculiar redox behavior [39]. Collectively, investigations on these minimal structural features reaffirmed the importance of electronic properties concerning aromatic fragments as a guiding motif in modulating Aβ aggregating processes.

### 2.2 Peptides

**Figure 3:**
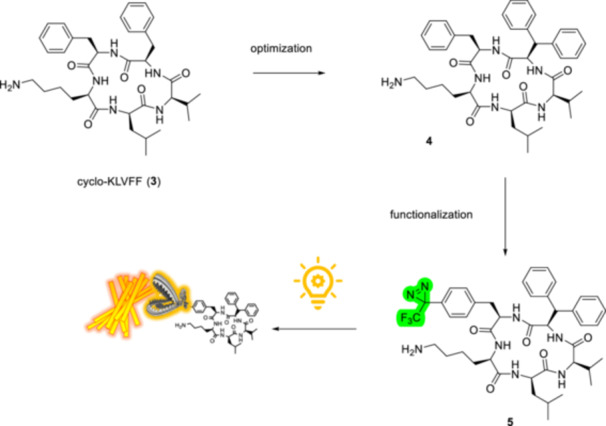
Peptides exerting Aβ antiaggregating activities based on a covalent mechanism.

The interest in peptide aggregation inhibitors originally started with the aim to identify the key structural regions involved in amyloid aggregation. Thus, several Aβ peptide fragments have been evaluated for the antiaggregating potential. Particularly, Lys‐Leu‐Val‐Phe‐Phe (KLVFF), corresponding to the Aβ16‐20 fragment, by strongly binding to the self‐recognition region of Aβ, moderately interfered with the Aβ42 fibrillization step [40]. Further optimizations led to the head‐to‐tail cyclic derivative **3** and its derivative **4**, able to reduce Aβ42 neurotoxicity at micromolar levels by lowering the aggregating capability of Aβ (Figure 3) [41]. A light‐controlled reactive diazirine was later inserted in the side chain of Phe5 residue to increase the target engagement of compound **4** (**5**, Figure 3) [42]. In this case, the covalent interaction upon irradiation occurred with Tyr10, triggering a significant decrease in both the amount of amyloidogenic cross‐β‐sheet structure and Aβ42‐induced toxicity.

### 2.3 Metals

Metal ions play an essential role in cellular physiology, and their dyshomeostasis is often related to pathological conditions. A high concentration of Fe, Zn, and Cu is detected in Aβ plaques, and these interactions trigger oxidative processes and modulate the aggregating pattern of Aβ [43]. Based on these premises, plenty of metal‐based ligands or metal chelators endowed with antiaggregating properties and antioxidant potential have been reported among the years [44, 45]. Particularly, metal complexes usually intervene by coordinating histidine or methionine residues through ligand exchange, causing reduced oligomer formation or stabilization of small oligomers as well as non‐aggregating prone, and non‐toxic high MW aggregates [46, 47, 48]. Transition metal complexes (*e.g*., Co, Rh, Ir, Pt, and Ru among others) proved to efficiently covalently bind Aβ after preliminary strong non‐covalent interactions between their hydrophobic ligands and the peptide, thus resulting in a valid source for potential therapeutics and diagnostics for neurodegenerative pathologies [49].

Furthermore, metal‐oxo clusters, commonly called polyoxometalates (POMs), proved to modulate Aβ42 aggregation through interaction with the cation cluster subregion, which is located next to the central hydrophobic region KLVFF. Therefore, to increase the specificity of action of POMs, a thiazolidinethione moiety has been inserted to covalently trap the vicinal Lys16 [50]. The obtained derivative not only covalently engaged Lys16 and subsequently inhibited Aβ aggregation but also proved in vitro and in vivo neuroprotective efficacies with confirmed blood–brain barrier (BBB) permeability. However, these represent only a glimpse of metal‐based antiamyloidogenic approaches [51, 52].

### 2.4 Penicillins

Another common approach in drug discovery campaigns consists of repurposing of marketed drugs, due to the already known safety profile and favorable PK properties. In this case, among drugs bearing reactive fragments, several antibiotics previously demonstrated neuroprotective and antiaggregating effects [53]. Particularly, the antiamyloidogenic mechanism of action of penicillins, exploiting benzylpenicillin as a test case, have been investigated in this respect [54]. Albeit not clarified yet the specific involved residue (*e.g*., serine's hydroxyl, lysine's amine or *N*‐terminus group), benzylpenicillin formed a covalent adduct with Aβ40 through the β‐lactam ring opening, resulting in lowered Aβ neurotoxicity and aggregation.

## 3 Tau Protein

Tau is a microtubule‐associated protein primarily localized in axons and responsible for axonal transport and microtubule stability, organization, and function. Tau directly interacts with tubulin through a microtubule‐binding domain (MBD) to orchestrate its assembly and stabilization into microtubules with consequent regulation of cytoskeleton plasticity. Under physiological conditions, tau is a highly soluble and natively unfolded protein with a random conformation in aqueous solution [55]. However, due to impaired post‐translational modifications (*e.g*., phosphorylation state and proteolytic processing), mutations or external stimuli, it dissociates from axonal microtubules, thus causing pathological axonal transport impairment and synaptic dysfunction. Furthermore, detached tau accumulates and promotes misfolding and self‐aggregation, leading to the formation of neurotoxic NFT [56, 57]. Normally, the aggregation process stems from the two highly aggregation‐prone fibril‐forming hexapeptide motifs ^275^VQIINK^280^ (PHF6*) and ^306^VQIVYK^311^ (PHF6), located within the four‐repeat (4 R) microtubule‐binding domain, which easily adopt cross‐β structures, acting as key nucleation sites for tau fibrillization [58]. At the molecular level, the starting point of this stepwise process is represented by a disulfide binding between Cys291 and Cys322 (*i.e*., both proximal to PHF6 and PHF6*), which results in dimer formation, followed by higher order oligomers produced sequentially [59]. At this step, the soluble oligomers grow and precipitate with a β‐sheet structure until obtaining insoluble paired helical filaments (PHFs) and then NFT [60]. The so‐formed aggregates are able to trigger a neurotoxic cascade characterizing an entire group of neurodegenerative disorders also known as tauopathies: defined as primary tauopathies if tau has a predominant effect on neurodegeneration (*e.g*., progressive nuclear palsy, corticobasal degeneration, Pick's disease, argyrophilic grain disease) or secondary where tau is not the unique pathological feature (*e.g*., AD) [61].

**Figure 4:**
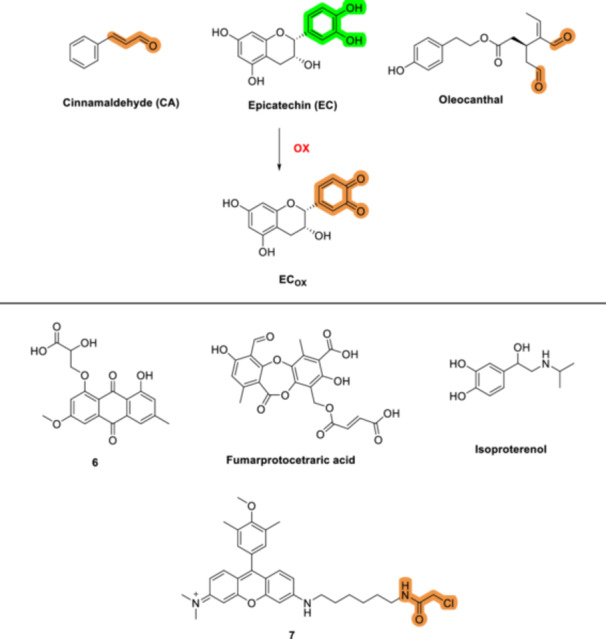
Natural products and derivatives exerting tau antiaggregating activities based on a covalent mechanism.

Among the plethora of different therapeutic approaches targeting tau, covalent aggregation inhibitors deserve an important mention, thanks to the presence and role played by the covalently vulnerable Cys291 and Cys322 in the aggregating processes [57, 62]. Even in this case, the research lines devoted toward the development of antiaggregating inhibitors mainly originated from the investigation on natural products and derivatives [63]. For example, from studies on the biological properties of cinnamon extract emerged cinnamaldehyde (CA) and epicatechin (EC) as two promising tau‐interacting compounds (Figure 4) [64]. The antiaggregating properties of both were evaluated exploiting the recombinant protein tau187 as a test model, which retains all full‐length tau characteristics and contains the part known to associate into the structured β‐sheet forming the core of tau tangles. Pre‐incubated tau with CA registered a significant reduction in the number of formed fibrillar structures analyzed by electron microscopy, and the reactivity of CA toward tau was attributed to its α,β‐unsaturated aldehyde moiety, which enables covalent modification of nucleophilic residues such as cysteines. Tau contains only two cysteine residues, both located within the MBD in the conserved SKCGS sequence. Experiments with tau187 demonstrated that two CA molecules associated with the protein in a time‐dependent manner, resulting in near‐complete masking of free thiol groups. However, the gradual recovery of thiol reactivity over time and glutathione competition indicated that the CA–tau interaction is reversible. Unambiguous confirmation of CA‐mediated inhibition via interaction with tau cysteines was obtained by comparing the different interacting behaviors using a synthetic SKCGS peptide and a tau187 double cysteine knockout mutant. Important to note that the formation of CA‐tau adduct preserved the normal tau function in microtubule assembly. Finally, the loss of effect observed with similar saturated aldehydes suggested that CA reacts with tau via a Michael addition between the cysteine's thiol and the β‐carbon of CA's α,β‐unsaturated system [64].

Differently, EC acted as a potent inhibitor of tau aggregation only upon oxidation, indicating that oxidized EC species (EC_OX_), likely quinone or quinone‐like products, are responsible for the inhibitory activity (Figure 4). Even in this case, the interaction of EC_OX_ with cysteine residues was confirmed by MS using the SKCGS peptide. MS/MS analysis demonstrated the formation of an EC_OX_–cysteine adduct, while excluding modification of amino groups, indicating selective reactivity toward thiol groups [64].

Furthermore, CA and EC also showed important effects in the prevention of cysteine oxidation, which accounts for the establishment of inter‐molecular disulfide bridges, thus leading to the formation of large complexes observed under oxidative stress conditions [64].

Also, the olive oil‐derivative (‐)oleocanthal (Figure 4) demonstrated to interfere with tau fibrillization processes [65]. Thanks to the dialdehyde moiety, it exhibited multiple nonspecific covalent interactions with tau‐441 (wild‐type tau protein), as detected by mass spectrometric analyses, inducing a conformational rearrangement of secondary structure from a random coil to an α‐helix, which accounts for its antifibrillogenic activity [65]. Further structural insights accounted for the covalent interaction with Lys311 via initial Schiff base formation as the essential step in reducing tau filament formation [66].

From lichens, other interesting nature‐derived products were obtained in this respect, such as the anthraquinone derivative **6**, and three other products sharing an α,β‐unsaturated carbonyl group, among which fumarprotocetraric acid resulted as the most active (Figure 4) [67, 68]. The lichen‐derived compounds were tested as tau aggregation inhibitors using maleimide cysteine labeling, which monitors tau aggregation via cysteine–cysteine interactions. After cysteine‐labeling and tau aggregation, both compounds reduced oligomer progression and the entire β‐sheet content, indicating direct interactions with cysteines and consequent interference with cysteine‐mediated tau oligomer assembly [67]. Further investigations are needed to clearly depict the exact mechanism of action at the molecular level. Similarly, several other compounds were reported over the years inhibiting tau aggregation through a covalent‐based mechanism, but mainly acting as cysteine pro‐oxidant agents [69] or interacting with cysteines through putative covalent interactions without the experimental elucidation of the covalent mechanism [70, 71, 72, 73]. A particular mention deserves the promising mitigation in tau oligomer formation of several catechol‐bearing compounds, reaffirming the important antiaggregating properties of this pro‐electrophile fragment [74]. Particularly, isoproterenol (Figure 4), also thanks to its BBB permeation profile, registered an important reduction in tau filaments and granular oligomers formation. The interaction with the target occurred at the level of the microtubule‐binding regions and relied on the presence of the free cysteine residues. Further MS investigations confirmed the formation of a covalent adduct at Cys. In an animal model of tauopathy, isoproterenol orally administered reduced insoluble tau aggregates and neuronal loss in the cortex and hippocampus, mitigating related brain dysfunction [74].

A different approach was followed to achieve thiol‐reactive rosamine‐based fluorescent derivatives [75]. Rosamine is a rhodamine derivative endowed with fluorescent properties and was functionalized with a chloroacetamide moiety, through the interposition of different linkers, to covalently trap fibrillization‐competent cysteine residues, thus enabling the direct visualization of fluorescently labeled tau species. Among different tau species, compound **7** (Figure 4), bearing a hexyl diamine linker, prominently labeled monomers and dimers, resulting in a strong inhibitory effect on tau aggregation (IC_50_ = 3.7 µM by ThS method) [75].

**Figure 5:**
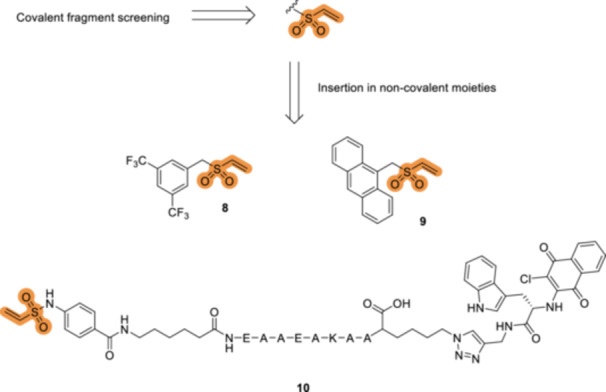
Development of covalent tau aggregation inhibitors from fragment screening to tau binders' optimization.

Finally, a rational strategy to obtain a covalent inhibitor of tau‐fibril formation was recently reported by Keserü et al. [76]. A preliminary warhead selection was carried out on a small library of covalent fragments, determining the labeling efficiency of a tau‐K18 construct with orthogonal methods (*i.e*., Ellman's assay, MS, and ^19^F NMR). Based on these results, the vinylsulfone group represented the best choice in terms of tau cysteines' labeling, reactivity, and aqueous solubility (Figure 5). Therefore, the vinylsulfone warhead was attached to different non‐covalent scaffolds (*e.g*., hydrophobic moiety, cholesterol‐, rhodamine‐, naphthoquinone‐tryptophan‐ and peptide‐based derivatives), which previously demonstrated the ability to anchor close to the PHF6* and PHF6 region of tau, to prioritize the non‐covalent interaction with the target and then facilitate the covalent engagement with Cys322 and Cys291 residues (Figure 5). All the designed inhibitors decreased the aggregation rate of tau‐K18 compared to the untreated ones, dependent on the presence of Cys residues, as demonstrated to the loss of activity with mutant tau‐K18 constructs (*i.e*., C322S and C291S). MS/MS proteomics proved the covalent engagement through a double labeling at both Cys for **8** and **9**, and a single adduct at Cys291 for **10** (Figure 5). With circular dichroism and transmission electron microscopy analyses, the developed tau binders confirmed induced conformational changes on tau morphology that resulted in decreased aggregate formation [76].

## 4 α‐Synuclein (α‐Syn)

α‐Syn is a 140‐residue protein predominantly located at the brain synaptic termination of dopaminergic neurons. Albeit its specific functions are still not completely understood, its localization and behavior under physiopathological conditions suggest a role in vesicle trafficking at synapsis [77]. α‐Syn is considered as a monomeric intrinsically disordered protein, although it can also bind to lipidic membranes through the *N*‐terminal domain in an α‐helical conformation [78]. In the presence of pathological triggers, α‐Syn acquires rigid β sheet‐enriched conformations that can further polymerize into neurotoxic oligomers and fibrils. In this case, the α‐Syn fibrillization process seems to stem from homomeric α‐Syn interactions between two sequences in the *N*‐terminal domain, which then leads to cross‐β formation [79]. Furthermore, the majority of missense mutations related to synucleinopathies affect this region, confirming the pivotal role of this fragment in the aggregation process [80]. Under physiological conditions, the α‐Syn multimers are removed by the protein degradation machinery, but if genetic or environmental factors (*e.g*., mutations and induced oxidative or post‐translational modifications) boost misfolding and fibrillization steps, then occur accumulation and redistribution of α‐Syn in various brain regions and cellular populations [81]. These changes in the nature and localization of α‐Syn, leading to neuronal and glial inclusions also called Lewy bodies, exert common pathogenic roles which characterize a group of brain disorders known as synucleinopathies, which include PD, Dementia with Lewy bodies (DLB), and multiple system atrophy (MSA) [82].

Several small molecules have been reported to modulate α‐Syn amyloid aggregation, and covalent inhibitors represent a small group mainly exploited to elucidate neurotoxicity properties of formed α‐Syn oligomers and fibrils [83]. Among natural polyphenols, flavonoids represent one of the most efficient modulators of α‐Syn misfolding and aggregation processes. Several SAR studies mainly correlate these antiamyloidogenic properties to hydrophobicity, number, and spatial disposition of aromatic hydroxyl groups with resulting oxidative properties in a similar way to what has been reported for Aβ [84]. For example, EGCG demonstrated to inhibit α‐Syn fibrillogenesis, disaggregate preformed fibrils, and transform fibrils into smaller non‐toxic aggregates by means of hydrophobic‐driven interactions and without any proof of covalent binding [85, 86]. Differently, the formation of an imine adduct between a lysine side chain of α‐Syn and the oxidized quinone ring of baicalein, or its glucuronide baicalin, accounted for the biological properties, becoming one of the most active fibrillization inhibitors [87, 88]. Particularly, following in‐depth MS investigations, Lys21 was highlighted as a potential residue involved in Schiff base formation or Tyr39 as an additional target for Michael addition with quinone ring [88]. Following this mechanism, baicalein turned out with a polyhedral profile spanning from the inhibition of α‐Syn oligomerization or stable oligomer formation, preventing α‐Syn oligomer toxicity [89]. Interestingly, a similar mechanism was also proposed regarding the amylin antiaggregating properties of baicalein [90].

**Figure 6:**
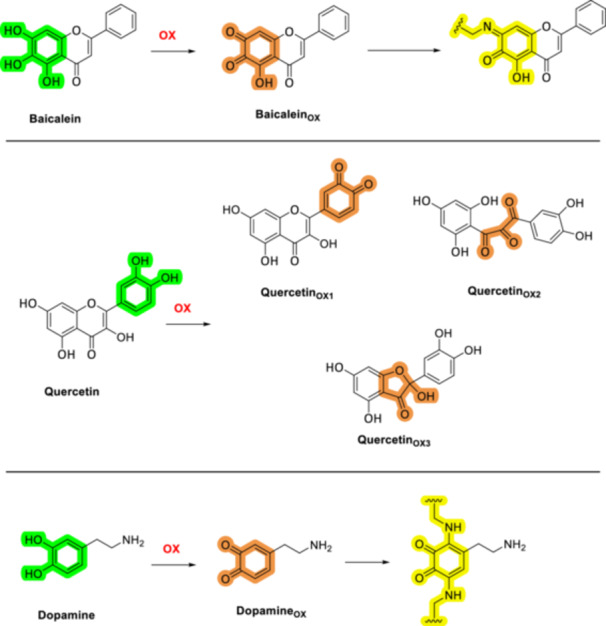
Natural products and derivatives exerting α‐Syn antiaggregating activities based on a covalent mechanism.

Analogously, quercetin covalently bound to α‐Syn in a 1:1 ratio, albeit the specific molecular mechanism has not been identified [91]. Upon adduct formation, quercetin inhibited α‐Syn fibrillization processes by reducing fibril growth and stabilizing formed oligomers. Furthermore, the main oxidation products of quercetin (Figure 6) resulted in the key players in the inhibitory mechanism, underlining the importance of oxidative activation into an electrophile for the resulting biological activity. In this case, it was proposed that formed quercetin‐α‐Syn adducts increased surface hydrophilicity, therefore preventing fibril formation and further aggregation.

Given the entangled neuropathogenesis of PD, both dopaminergic deficits and α‐Syn misfolding processes are strictly interconnected. Particularly, while α‐Syn impaired dopamine neurotransmission at multiple levels, dopamine and its precursors/by‐products demonstrated to affect the amyloidogenic pathway [86, 92]. At the molecular level, dopamine forms covalent adducts with α‐Syn's lysine residues upon oxidative activation into the quinone form, resulting in a complete inhibition of α‐Syn fibrillization. However, cross‐linked α‐Syn‐dopamine products, mainly monomers and oligomers, maintained toxicity effects, thus confirming early‐stage aggregates are in charge of neurotoxicity more than fibrils [93]. Nevertheless, the pro‐oxidant activity of dopamine was also accounted for the antiamyloidogenic properties through the oxidation of α‐Syn methionine residues, which leads to lowered fibrillogenesis propensity for oxidized α‐Syn [94].

Several other (pro)electrophilic compounds have been accounted for α‐Syn antiaggregating properties, but without detecting a covalent mechanism of action [83].

## 5 Superoxide Dismutase 1

**Figure 7:**
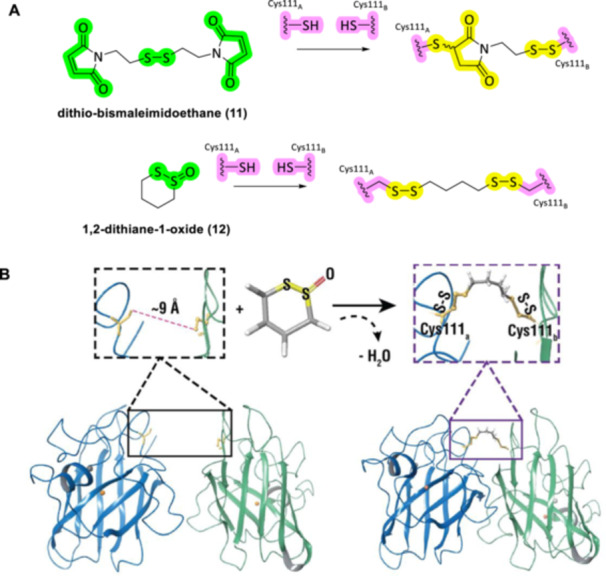
Compounds exerting SOD1 antiaggregating activities based on a covalent mechanism (1). (A) Cross‐linking agents and (B) crystal structure of SOD1, highlighting opposing Cys111 residues on both monomer A (blue) and monomer B (green) with a representation of the **12** cross‐linked SOD1 dimer. Cu and Zn molecules are represented by orange and gray spheres, respectively. Adapted from Hossain, M. A.; Sarin, R.; Donnelly, D. P.; Miller, B. C.; Weiss, A.; McAlary, L.; Antonyuk, S. V.; Salisbury, J. P.; Amin, J.; Conway, J. B.; et al. Evaluating protein cross‐linking as a therapeutic strategy to stabilize SOD1 variants in a mouse model of familial ALS. PLoS Biol. 2024, 22 (1), e3002462. DOI: 10.1371/journal. pbio.3002462 [100]. Licensed under CC BY 4.0.

Functionally, SOD1 is an antioxidant enzyme present in cytosol, outer‐mitochondrial membrane, and intermembrane space of mitochondria with the duty to eliminate superoxide ions through disproportionation, thus protecting cells from oxidative damage [95]. At the physiological level, SOD1 is a stable homo‐dimer, while ALS is usually related to mutated variant SOD1 isoforms, which are more inclined to dimer dissociation, thus triggering misfolding and aggregation of resulting monomers into neurotoxic species [96]. For these reasons, stabilizing SOD1 homodimer and preventing aggregate formation was proposed as a potential therapeutic strategy for years. Besides pathological mutations, the stability of the homodimer isoform depends on the presence of one zinc and one copper ions as well as one disulfide intramolecular bond (*i.e*., Cys57‐Cys146) for each subunit [97]. Particularly, the removal of these ions or disulfide bond disruption promote SOD1 fibrillization. The presence of the other two free cysteines (*i.e*., Cys6 partially accessible and Cys111 solvent exposed) emerged with particular interest for potential electrophilic targeting approaches. Interestingly, the covalent modulation of Cys111 residues by means of a single reactive small molecule or cross‐linking them through the dimer interface groove resulted in effective alteration of the amyloidogenic process. To note, the oxidative modification of Cys111 under pathological conditions promote SOD1 aggregation independently from the intermolecular disulfide bond that stabilizes toxic clusters, underlining its key role in homodimer stabilization [98]. The proximity of two Cys111 residues across the dimer interface (*i.e*., ~10 Å) was first exploited to stabilize the SOD1 dimer by means of different cross‐linking agents bearing a disulfide bridge [99]. In the case of dithio‐bismaleimidoethane, the cross‐linking between two subunits occurred through the interposition of a thiomaleimidoethane linker with one Michael addition on α,β‐unsatured carbonyl of maleimide from one Cys111 and thiol‐disulfide exchange from the other (**11**, Figure 7A). After this chemical modification, the SOD1 dimer turned out with higher stability, and some fALS inactive mutants restored its biological activity [99]. More recently, the same strategy was tested for its in vivo efficacy by employing cyclic thiosulfinate derivatives [100]. After cross‐linking SOD1 dimer, 1,2‐dithiane‐1‐oxide (**12**, Figure 7A,B) stabilized multiple fALS variants both at the cellular and animal levels. Notably, compound **12** administered at 30 mg/kg via sc in mice bearing the G93A variant showed 86% conversion of SOD1 to cross‐linked dimer in the brain after only 1h‐treatment. Unfortunately, two different fALS SOD1 mouse variants **12**‐treated showed from modest to no survival benefit, thus requiring further optimizations [100].

**Figure 8:**
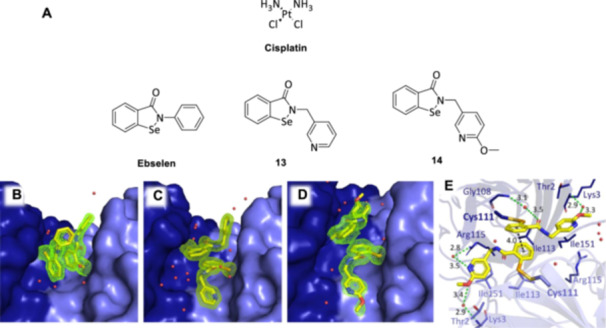
Compounds exerting SOD1 antiaggregating activities based on a covalent mechanism (2). (A) Cisplatin, ebselen, and its derivatives. Co‐crystalised structures of SOD1 with ebselen (B), **13** (C), and **14** (D). Individual SOD1 monomers are colored in dark and light blue surface. (E) Binding mode of **14** is shown among amino acid residues (dark and light blue sticks) and water molecules in the SOD1 dimer interface. Hydrogen bonds and distances between molecules are shown as green and black dashes, respectively. Numbers represent distances in Å. Adapted from Watanabe, S.; Amporndanai, K.; Awais, R.; Latham, C.; Awais, M.; O'Neill, P. M.; Yamanaka, K.; Hasnain, S. S. Ebselen analogs delay disease onset and its course in fALS by on‐target SOD‐1 engagement. Sci. Rep. 2024, 14 (1), 12118. DOI: 10.1038/s41598‐024‐62903‐5 [101]. Licensed under CC BY 4.0.

Among non‐cross‐linking agents, cisplatin (Figure 8A) was one of the first small molecules proved to bind Cys111 (*K_d_* = 37 ± 3 µM for apo form) and stabilize the dimer without directly tethering the two subunits [102]. With a 1:1 interacting ratio for each subunit, cisplatin blocked cysteines for amyloidogenic intermolecular disulfide bond formation, thus preventing the oligomerization step. Furthermore, due to its intrinsic reactivity, cisplatin was able to split the already formed disulfide bridges in oligomers and therefore dissolve the preformed aggregates. Due to this promising profile, other less toxic and cysteine‐sensitive compounds were evaluated in this respect. From a crystallographic screening on cysteine‐reactive compounds, ebselen (Figure 8A) and ebsulfur were able to covalently engage Cys111 of SOD1 without affecting Cys6 and the disulfide bond Cys57‐Cys146 [103]. Both of them underwent nucleophilic attack of the free sulfhydryl group, forming a disulfide or selenylsulfide bridge after opening of the heterocyclic ring. The resulting interposition of two “opened molecules” among the dimer groove docked at two Cys111 residues significantly stabilizes the formed dimer, also thanks to additive inter‐dimer pi‐pi stacking or hydrogen bonds (Figure 8B). These tridimensional arrangements contributed to increased or restored dimer formation in different pathological SOD1 mutants without interfering with the physiological interaction of SOD1 with the human copper chaperone for SOD1 (*h*CCS). Unfortunately, this mechanism can be maintained only in low‐reducing cellular environments, because the selenylsulfide bond is easily cleaved by cellular thiols. Furthermore, at the cellular level, ebselen demonstrated to stimulate correct SOD1 maturation by favoring the intra‐disulfide bridge thanks to its oxidizing properties: a transient selenylsulfide bond is rearranged by thiol‐selenyl exchange, yielding SOD1 disulfide. Due to this polyhedric activity, the stabilization of mutant SOD1 from ebselen was confirmed also in cells [103]. In SOD1 transgenic mice, ebselen administered at 24 mg/kg *per os* delayed disease onset without affecting survival time [104]. To improve its therapeutic and physicochemical properties, different phenyl and benzyl substituents were evaluated on benzoselenazol‐3‐one core. Different from ebselen and *N*‐aryl derivatives, *N*‐benzyl ones are arranged in perpendicular disposition due to their higher flexibility. Particularly, compounds **13** and **14** bearing a pyridyl ring (Figure 8A–E) exerted strong dimer stabilizing profiles by establishing stable cross‐monomer linking interactions with Asp109. Furthermore, the same derivatives remained the only showing neuroprotective activity until 10 µM in mouse neuronal cells transfected with SOD1 fALS mutants [104]. In human neuroglioma cells, the same neuroprotective profiles were confirmed, albeit requiring higher dosages. Regarding the antiaggregating profile toward four different fALS SOD1 mutants, compound **14** outperformed both ebselen and **13** in terms of reduced number and dimension of cellular inclusions while restoring a phenotype similar to a wild‐type one. In the same animal model and dosage, **14** slightly increased the delay of disease onset without a significant extension of the survival profile. Furthermore, in vivo **14** treatment reduced the accumulation of misfolded SOD1, oligomers, and SOD1 insoluble aggregates, protecting the neuromuscular junction from pathological muscle denervation and therefore validating this first animal proof‐of‐concept, albeit requiring further optimization for ameliorating the PK profile [101].

## 6 Conclusions

Direct inhibition of misfolding and aggregation processes still remains a tricky and underexplored strategy in neurodegenerative proteinopathies, mainly because of the claimed undruggability of related disordered proteins. In this respect, the chance to covalently trap the targeted protein deserves further considerations both as a means for elucidating at the molecular level these pathological mechanisms or for reconsidering it as a potential therapeutic tactic. However, pathologically activated disordered proteins remain difficult to target due to the lack of stable and well‐defined binding sites, while exploiting covalent binders to this aim is usually related to low selectivity due to the presence of similar β‐sheet structures among different monomers. Therefore, the opportunity to address unique functional nucleophilic residues with tailored covalent modulators may prove to be a worthy avenue for mitigating weak affinity issues.

Polyphenol derivatives are absolutely predominant among covalent modulators herein reported. Thanks to their peculiar proelectrophilic moieties, they can be considered as natural pro‐drugs able to exert covalent inhibition only upon oxidative activation (*i.e*., autoxidation or induced from pathological oxidative stress conditions) into their electrophilic counterparts. As a confirmation of facts, antiaggregant effects of such polyphenols are often related to the corresponding antioxidant/electronic properties [37, 88, 105]. Furthermore, the covalent interaction usually occurs with nucleophilic residues upon proper accommodation into specific hydrophobic pockets, under the scheme of a rough targeted covalent inhibitor's mechanism of action. However, polyphenols feature major PK drawbacks that require relevant considerations.

In other cases, selectivity of action can be achieved by addressing the structural peculiarity of the protein (*e.g*., cross‐linking Cys111 in SOD1), which, however, demands a prior deep understanding at the molecular level of misfolding and aggregation processes.

The majority of the above‐discussed inhibitors were mainly identified after a serendipitous discovery or repurposing of natural compounds, pointing out vulnerabilities and challenges in adopting a rational approach targeting disordered proteins. Differently, among non‐covalent modulators, some ligand‐ or target‐based designed inhibitors were successfully developed [106, 107, 108, 109]. However, to this aim, a covalent‐first approach can be more appropriate and has already demonstrated its suitability, as reported by Keserü et al. [76], by exploiting different instrumental analyses and overcoming phenotypic or high‐throughput screenings.

Identifying valid covalent ligands can also represent the starting point for developing new chemical modalities that can broaden the toolbox exploitable to investigate misfolding and aggregating processes at the cellular level. For example, following the surge of proximity inducers, several degraders were reported to efficiently target and degrade intracellular amyloid proteins via the ubiquitin‐proteasome system [110]. Notably, similar degradation strategies are also being developed toward extracellular neurotoxic aggregates [111]. In this context, despite a single turnover degradation, degraders built on irreversible ligands of the target protein can enhance selectivity and potency [112]. Furthermore, degrader chemical probes binding covalently to the target protein can allow to degrade proteins difficult to target, as is perfectly the case of misfolded proteins for all the reasons reported above. Exploiting the same principle for targeting the undruggable, covalent ligands can be leveraged to develop functional probes as amyloid imaging sensors or activity/affinity‐based probes for aggregated proteome profiling [113, 114, 115]. The superior selectivity of covalent‐type tools in trapping reactive nucleophiles only in specific folding conformations that restrict their accessibility/exposure may be crucial to track and visualize the misfolded and aggregated proteome in the complex cellular environment [116]. In this framework, proximity labeling technologies through induced covalent modulation of the target protein are going to be the next precision tools to finely map neurodegenerative interactomes of misfolded aggregates [117, 118, 119]. Beside exploring composition, distribution and related cellular networks, a class of covalent modifiers emerged as prospective therapeutics by further inhibiting amyloid aggregation and favoring aggregates clearance upon catalytic modification [110, 120]. Collectively, these examples represent only a glimpse of the prospective capabilities of covalent modulators for advancing next‐generation chemical modalities.

Targeting misfolding and aggregation processes through direct interaction with monomers, thus preventing oligomers formation, is still an approach to pursue, and a covalent strategy can boost this battle plan. Albeit a potential clinical translation is still far away, covalent modulators have been demonstrated to provide valuable tools to investigate at the molecular level the structural motifs guiding these assemblies and the caused pathological processes. As we gain more details about key interactions regulating neurotoxic folding/aggregation, we will be closer to efficiently develop small molecules able to modify disordered protein's properties in ways that are useful for drug discovery.

## Conflicts of Interest

The authors declare no conflicts of interest.

## Data Availability

Data sharing is not applicable to this article as no datasets were generated or analyzed during the current study.
